# Lifestyle Health Behaviors of Nurses and Midwives: The ‘Fit for the Future’ Study

**DOI:** 10.3390/ijerph15050945

**Published:** 2018-05-09

**Authors:** Lin Perry, Xiaoyue Xu, Robyn Gallagher, Rachel Nicholls, David Sibbritt, Christine Duffield

**Affiliations:** 1 Faculty of Health, University of Technology Sydney, Ultimo 2007, Australia; Xiaoyue.xu@uts.edu.au (X.X.); David.Sibbritt@uts.edu.au (D.S.); 2 Charles Perkins Centre, Sydney Nursing School, University of Sydney, Camperdown 2006, Australia; Robyn.Gallagher@sydney.edu.au; 3 Cancer Society of New Zealand, Wellington 6022, New Zealand; rachel@cancer.org.nz; 4 Faculty of Health, Edith Cowan University, Perth 6027, Australia; Christine.Duffield@uts.edu.au

**Keywords:** health behaviors, health promotion, nursing, midwifery, workforce, non-communicable diseases, lifestyle, ageing

## Abstract

Nurses and midwives (nurses) are the principle role models and health educators for the wider population. This study sought to identify the health-related behaviors of the nursing workforce of New South Wales (NSW), Australia, compared to contemporary recommendations for healthy living and to the Australian general population, matched by gender and age. An electronic cross-sectional survey delivered in 2014–2015 recruited 5041 nurses through the NSW Nurses and Midwives Association and professional networks. Validated health behavior measures were collected and compared to Australian National Health Survey data. Compared with younger nurses, older nurses reported greater adherence to fruit and vegetable guideline recommendations, but were more likely to be overweight or obese. Younger nurses (25–34 years) had the highest risk of harmful drinking. Compared with the Australian general population, slightly higher percentages of nurses met dietary recommendations and slightly fewer were obese, had central adiposity or smoked. Nurses had lower physical activity levels and higher levels of risky drinking across most gender and age groups. Many nurses have lifestyle health behaviors that place them at high risk for developing non-communicable diseases, sometimes at higher risk than the Australian population to whom they deliver health education. Health promotion strategies for nurses are urgently required.

## 1. Introduction

Population ageing is a worldwide phenomenon which is leading to a shift from communicable to non-communicable diseases (NCDs) and their associated disabilities [1,2]. NCDs are by far the leading cause of death in the world, killing more than 36 million people or 63% of all deaths annually [3]. In most developed countries, NCDs are also the leading cause of illness and disability; in Australia in 2007–8, for example, one in fifty people reported four or more chronic health conditions. This growing burden of NCDs is reducing workplace productivity, decreasing rates of workforce participation and limiting economic growth [4]. Compared to people without NCDs, people with three or more NCDs are only about half as likely to be in the paid workforce. This poses major public policy challenges that impact spending in the health system [4,5].

The increasing prevalence of NCDs and their associated burden of ill health have been attributed to the advancing age of the population, to lifestyle changes and increased exposure to health risk factors [6]. Most NCDs are preventable, with the World Health Organization (WHO) estimating that at least 80% of all heart disease, stroke and diabetes is avoidable [4]. The major identified risk factors for NCDs are unhealthy diet, physical inactivity, obesity, raised lipids and tobacco use [7].

Diet has been known for years to play an important role in the prevention of NCDs, including cardiovascular disease, type 2 diabetes and cancer [7,8]. Internationally, economic growth, modernization, urbanization and globalization of the agri-food system have driven changes in eating habits and an accompanying increase in obesity and nutrition-related NCDs. Strong evidence has shown that adequate fruit and vegetable consumption, particularly, can reduce the risk and prevent the development of NCDs [9]. Similar environmental changes have led to physical inactivity, another major contributor to many NCDs. Participating in physical activity can prevent or minimize the risk of osteoporosis and help manage biomedical risk factors such as high cholesterol, high blood pressure and body weight [10]. Numerous studies have shown synergistic effects of combining physical activity with healthy eating patterns in preventing NCDs [7] and overweight/obesity. Overweight and obesity can be considered chronic conditions, as well as important biological risk factors for NCDs; now at epidemic proportions globally, at least 2.8 million people die every year as a result [1].

Other important behavioral risks include tobacco smoking, which is an important preventable cause of illness and death through diseases such as lung cancer, chronic obstructive pulmonary diseases and heart disease [10]. After years of debate on the relationship between alcohol drinking and NCDs, there is now consensus that moderate alcohol consumption (no more than two standard drinks/day) can reduce the risk of coronary heart disease, while higher risk drinking can contribute to development of high blood pressure, cardiovascular disease, type 2 diabetes, liver and kidney diseases [11].

Many studies have shown the benefit in disease prevention of adherence to recommendations for health behaviors: for example, large population studies have demonstrated that adherence to diet, exercise and alcohol recommendations lowered the risk of developing NCDs such as cancer and cardiovascular diseases as well as lowering premature mortality [12,13]. Nurses are also members of these communities; these population effects suggest potential benefits for the nursing workforce. The Australian nursing and midwifery workforce (subsequently referred to as nurses) is ageing faster than the Australian general population; 47% of Australian nurses are now aged 50 years and older [14]. The rapid ageing of this workforce has implications for their health, for a potentially substantial impact from an increasing burden of NCDs. With nurses’ health linked to intention to leave, this has implications for workforce retention [15].

There are also implications for the Australian community. Health education and health promotion have been identified as intrinsic to nursing [16,17], with the International Council of Nursing claiming a key role for the profession in promotion of positive lifestyles [18]. That nurses have a responsibility as role models for the community is a contentious view for some [19] but there is little challenge to the presumption that health professionals’ behaviors are an influence on the population [20].

Although the association between health behaviors and NCDs is well established, little information is available about nurses’ health behaviors and therefore their potential health risk. We previously explored the health behaviors of nurses of two Australian metropolitan inner-city hospitals, revealing high levels of NCD risk through high levels of overweight and obesity, risky drinking, and poor fruit and vegetable intake [21]. To better understand the health behaviors of this workforce, we conducted a larger study to: (1) identify the health-related behaviors of nurses in New South Wales (NSW) compared to contemporary recommendations for healthy living; and (2) compare nurses’ health-related behaviors to those of the Australian general population, matched by age and gender. 

## 2. Materials and Methods

A cross-sectional survey design was employed to collect data on nurses’ health and health-related behaviors. With the approval of the university Human Research Ethics Committee (UTS HREC 2013000741), a web-based questionnaire launched as part of the ‘Fit for the Future’ study [15] between June 2014 and February 2015 was delivered as a link in an email to the membership of the NSW Nurses and Midwives Association (the nursing and midwifery industrial body and professional organization of NSW) and an invitation was published in the Association journal. Snowballing through the research team’s professional contacts was also used.

### 2.1. Sample

Full details of methods are available [22]. Of the total 5446 responses, 405 (7.4%) were excluded due to missing data or because respondents were non-practicing. In total, 5041 nurses were included in the analysis.

### 2.2. Measures

Measures were derived from previously validated instruments and items [22]. Health-related behaviors reported in this paper are: vegetable, fruit, milk, ‘fast (or ‘junk’) food’ and soft drink consumption, physical activity level, Body Mass Index (BMI) and Waist Circumference (WC), risky drinking and tobacco smoking status.

Dietary intakes were measured by asking how many serves of vegetables/pieces of fruit/types of milk participants usually consumed each day; frequency and amounts of fast food and soft drinks. Physical activity was calculated by summing all the time spent walking briskly or doing moderate or vigorous leisure activity in the previous week. Weight, height and waist circumferences were self-reported. Body Mass Index (BMI) was calculated as weight (kg)/height (m^2^) and categorized based on the WHO criteria as underweight (BMI < 18.5 kg/m^2^), normal (18.5–24.99 kg/m^2^), overweight (25.0–29.99 kg/m^2^), and obesity (≥30.0 kg/m^2^) [7]. A waist measurement of 94 cm or more (for men) and 80 cm or more (for women) is identified in the Australian National Health and Medical Research Council guideline as indicative of increased risk of chronic diseases [23]. Alcohol consumption was identified by asking participants how often they drank; how many drinks (units of alcohol) they usually consumed on a day when they drank, and how often they had more than four drinks on one occasion. Risky drinking was defined as consuming more than two standard drinks per day on average or more than four on a single occasion at least once a month [24]. Current smokers included those who reported smoking daily, weekly and less than weekly.

### 2.3. Analysis

Vegetable and fruit intakes, physical activity levels and risky drinking were compared to relevant national recommendations and analyzed according to gender and age groups using Chi square tests. Analysis of variance (ANOVA) tests and logistic regression were used to assess significant associations between those who did or did not meet health behavior guidelines by gender and age. Comparisons were also made between sample findings and those of the Australian general population derived from the Australian National Health Survey of 2014–2015 [25]. As the number of nurses in the age groups 18–24 years (*N* = 143, 3.2%) and ≥65 years (*N* = 152, 3.4%) were small, we excluded these two age groups from comparisons with the Australian general population. Nurses were grouped by ages 25–34 years, 35–44 years, 45–54 years and 55–64 years for comparison with the Australian general population. Proportion tests were used to compare the proportional differences across age groups between NSW nurses and Australian general population. Data were analyzed using the Statistical/data Package STATA/SE 13.1 (STATA; StataCorp, Texas, TX, USA).

## 3. Results

The sample of 5041 nurses had a mean age of 48 (standard deviation (SD) = 11.4) years, with more than half aged 50 years and older (Table 1). Most (90.6%) were female and had at least a bachelor degree (72.2%). Two thirds lived in metropolitan areas, and most (71.7%) worked as foundational nurses (front-line clinical staff), most often (59.6%) in hospitals. Whilst the mean working week reported was 34.3 (9.8) h, 39.8% worked more than 40 h.

### 3.1. Nurses’ Health-Related Behaviors

#### 3.1.1. Diet

Adequate vegetable and fruit intakes were defined as five and two serves or more per day, respectively, based on the Australian Dietary Guidelines [26]. Only 8% (7.6% female and 0.4% male) of nurses met both these recommendations; more female than male nurses met the fruit recommendation (55.2% vs. 46.2%, *p <* 0.001); 11.5% of female and 5.2% of male nurses met the vegetable recommendation (*p <* 0.001). Significant differences were found in adherence to vegetable and fruit intake recommendations by age group (*p =* 0.001), with the youngest group (aged 25–34 years) less and the older group more adherent (Table 2).

Overall, 4.3% of female and 5.2% of male nurses did not drink milk. Among those who reported drinking milk, full fat milk was consumed by 25.9% of female and 38.7% of male whilst other types of milk (reduced/fat free/soya milk) was chosen by 74.1% female and 64.1% male nurses. Female nurses were significantly less likely to choose full fat milk, and more likely to choose other types of milk (*p <* 0.001).

‘Fast’ foods were not consumed by 28% of female and 17.5% of male nurses; 53.9% of female and 52.8% of male nurses ate fast foods less often than weekly; 17.6% of female and 29.7% of male nurses had fast food weekly or more frequently. Carbonated soft drinks were not consumed by 57.2% of female and 34.5% of male nurses; 34.4% of female and 49.4% of male nurses had ≤5 cups of soft drinks per week; 8.2% of female and 15.8% of male nurses drank more soft drinks than this per week. Male nurses had significant higher frequency and volume of consumption of fast foods and soft drinks than female nurses (*p <* 0.001). Young nurses (aged 25–34 years) ate fast food more frequently and drank more soft drinks than older nurses (*p <* 0.001).

#### 3.1.2. Physical Activity

Recommendations for minimum activity levels internationally and in Australia are to accumulate 150 to 300 min (2½ to 5 h) of moderate intensity physical activity or 75 to 150 min (1¼ to 2½ h) of vigorous intensity physical activity, or an equivalent combination of both moderate and vigorous activities, each week [27]. Almost half of all nurses (46.6%), but more male than female nurses, met the physical activity recommendations (51.5% vs. 46.0%, *p =* 0.03). No statistically significant differences were found in different age groups for those meeting the guideline, but male nurses were more likely than female nurses to do so (*p <* 0.02).

#### 3.1.3. BMI and Waist Circumference

Overall, 61% of nurses were overweight or obese: 31.3% were overweight and 29.7% obese. Mean (SD) BMI was 27.8 (6.3) kg/m^2^ for females and 27.9 (5.3) kg/m^2^ for males. A greater proportion of male compared to female nurses were overweight (46.1% vs. 29.6%, *p <* 0.001) but a greater proportion of female than male nurses were obese (30.2% vs. 25.1%, *p <* 0.001).

Mean waist circumference (WC) was 81.8 cm for female and 93.5 cm for male nurses. A greater proportion of female than male nurses had WC measurements that indicated risk of developing NCDs (66.0% vs. 42.1%, *p <* 0.001). Compared with the youngest age group (aged 25–34 years), nurses at older ages were significantly more likely to be overweight or obese (*p <* 0.001). Nurses at older ages were also significantly more likely to have central adiposity that put them at risk (*p <* 0.001). Compared with female nurses, male nurses were 64% more likely to be overweight or obese, but less likely to have central adiposity (Table 2).

#### 3.1.4. Risky Drinking

Risky drinking habits were reported by 16.2% of nurses. A greater proportion of male than female nurses reported risky drinking patterns (27.7% vs. 15.5%, *p <* 0.001). Significant differences were found between proportions with risky drinking across age groups (*p <* 0.001), with nurses in the youngest age group with highest proportions reporting ‘at risk’ behaviors, and male nurses with significantly higher rates of risky drinking than females (Table 2).

#### 3.1.5. Smoking

Internationally, health recommendations are for abstention from smoking. Our results showed that of 4529 responders, 10.3% self-reported current smoking (9.9% of female and 12.9% of male nurses, *p =* 0.05); 8.8% of nurses were daily smokers (8.6% female and 10.8% male nurses, *p =* 0.001); 1.5% of nurses smoked at least weekly (1.3% female and 2.1% male nurses, *p =* 0.001). 

Significant differences were found between daily smoking rates by age groups, with older nurses more likely to smoke daily. Male nurses were more likely to smoke daily than female nurses (*p <* 0.001) (Table 2). 

### 3.2. Comparing Health-Related Behaviors of Nurses and the Australian General Population

#### 3.2.1. Diet

**Figure 1:**
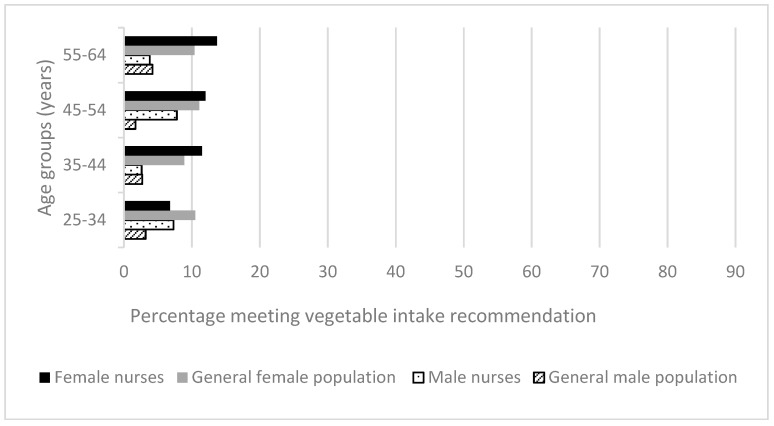
Health-related behaviors of nurses and midwives compared to the Australian general population 2014–2015 [28]: Vegetable intake.

**Figure 2:**
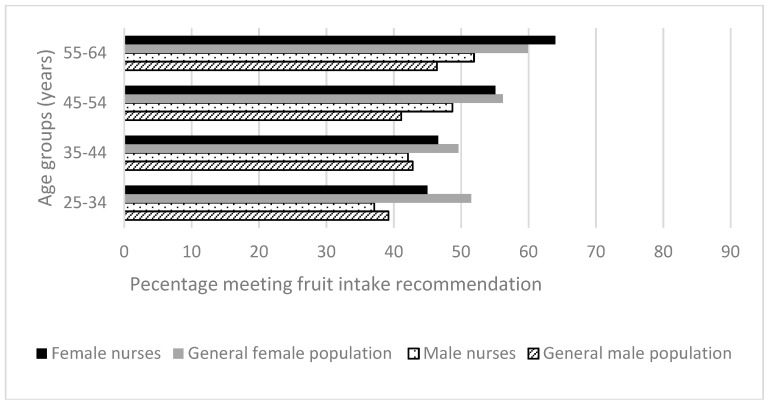
Health-related behaviors of nurses and midwives compared to the Australian general population 2014–2015 [28]: Fruit intake.

One in twenty (5.1%) members of the Australian population met the recommendations for vegetable and fruit intake [28], compared to 8% of nurses (Figure 1 and Figure 2). For both groups, far greater proportions of people met the fruit than vegetable consumption recommendation. Older age groups had healthier eating behaviors, except for older male nurses in the age groups 35–44 and 55–64 years, of whom only 2.6% and 3.8% met vegetable intake recommendations. However, these groups only numbered seven nurses in total.

Consistently, greater proportions of female nurses than female members of the Australian general population met the vegetable consumption recommendation, but this was not significant. A different pattern was seen for fruit consumption for most age groups: in those aged 25–34 years (51.5% of general female population vs. 45% of female nurses; *p =* 0.01), 35–44 years (49.6% of general female population vs. 46.6% of female nurses, *p =* 0.19) and 45–54 years (56.2% of general female population vs. 55.1% of female nurses, *p =* 0.56). In 8 of the 16 comparisons (age groups by gender, fruit and vegetable consumption) nurses demonstrated greater adherence with these dietary recommendations than the Australian general population. However, in most age/gender categories the differences between the adherence of the nurses and the relevant matched general population groups was small, averaging 3.2%, range 0.1% to 7.6%.

Overall, nurses were less likely to consume full fat and more likely to consume reduced fat/fat free/soy milk than the Australian general population. Comparing nurses’ milk consumption patterns to those of the Australian general population in the 2007–08 National Health Survey (excluding those who did not drink milk) revealed 42% of female Australians aged 15 years and over versus 28.4% of female nurses, and 54% of the male population versus 42.7% of nurses opted for full fat milk. Other types of milk (reduced fat/no fat/soy milk) were the choice for approximately 57% of the female population versus 71.7% of female nurses; of 46% of the male population versus 57.3% of male nurses [10]. Australian population data were not available by age. No comparable data were available for fast food or soft drink consumption.

#### 3.2.2. Physical Activity

**Figure 3:**
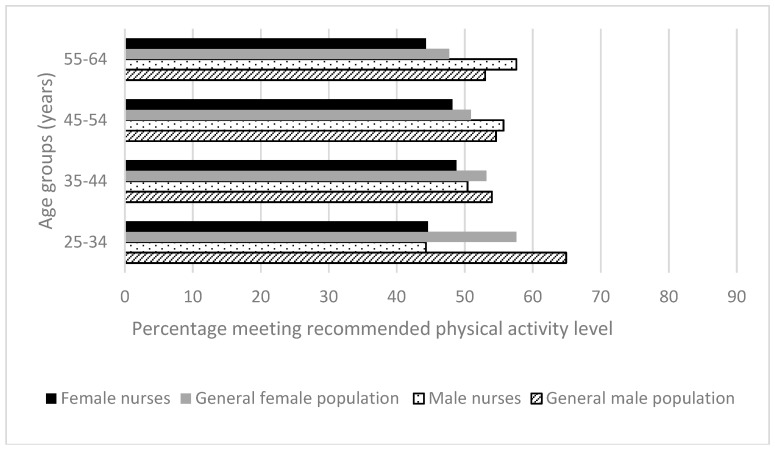
Health-related behaviors of nurses and midwives compared to the Australian general population 2014–2015 [29]: Physical activity.

Figure 3 shows that for all age groups of both genders, except for the two small older male groups, nurses had lower physical activity levels than the Australian general population [29] across gender and age groups, in particular for female nurses. The biggest difference was in the youngest age group, where 20.6% fewer males who were nurses than general population members met the physical activity recommendation (*p <* 0.001). For the 35–44 years age group, the difference between male nurses and general population members’ guideline adherence was 3.6% (*p =* 0.45), with the male nurses of the two older groups (aged 45–54 and 55–64 years) showing non-significantly greater adherence. For the female groups, the differences between nurses and the general population were non-significant at 13%, 4.4%, 2.7% and 3.4% (from youngest to oldest groups).

#### 3.2.3. BMI and Waist Circumference

**Figure 4:**
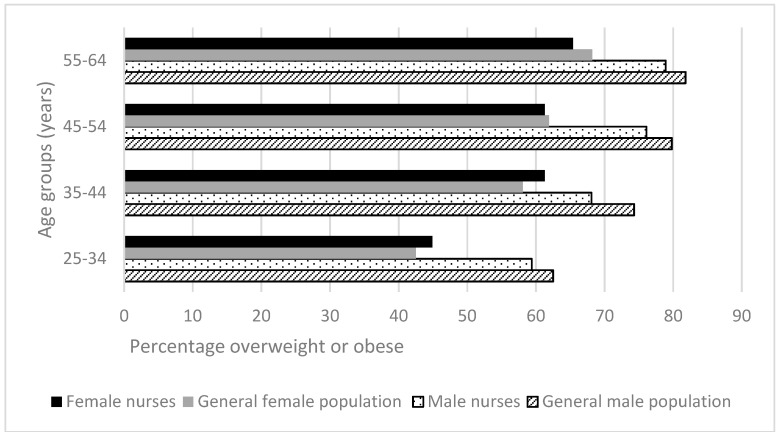
Health-related behaviors of nurses and midwives compared to the Australian general population 2014–2015 [25]: Body Mass Index (BMI) in overweight/obesity range.

**Figure 5:**
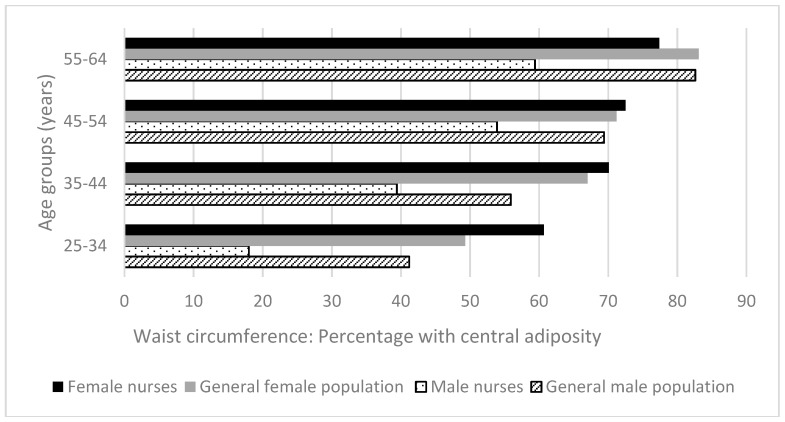
Health-related behaviors of nurses and midwives compared to the Australian general population 2014–2015 [25]: Waist circumference in central adiposity range.

A greater proportion of males than females were overweight and obese (Figure 4), while a greater proportion of females than males had central adiposity in both the general population [25] and nurses (Figure 5).

Compared to the general population, a slightly lower proportion of nurses were overweight or obese. In three of eight age/gender comparisons between nurses and the general population, nurses were more affected by overweight/obesity; in three of eight such comparisons, nurses were more affected by central adiposity. Differences between proportions of overweight/obesity in the nurse and general population matched groups were generally small, averaging 3.1% and ranging 0.6 % to 6.2% (*p* > 0.14). Similarities were seen for central adiposity in female groups, except for the youngest groups, where female nurses were significantly more often at risk (11.4% more, *p <* 0.001). The differences were more pronounced between the male groups; at all ages nurses fared better (average difference 19.6%, range 15.5% to 23.2%, *p <* 0.001).

#### 3.2.4. Risky Drinking

**Figure 6:**
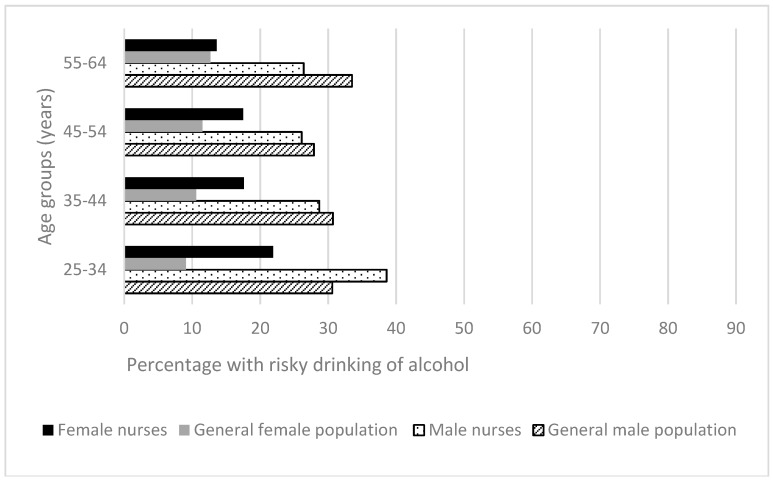
Health-related behaviors of nurses and midwives compared to the Australian general population 2014–2015 [24]: Risky drinking of alcohol.

In both nurses and the general population rates of risky drinking were higher amongst males than females. More than one third (38.6%) of male nurses and one in four female nurses aged 25–34 years reported risky drinking (Figure 6). For females of all age groups and for the youngest group of males, nurses had markedly higher proportions than the Australian general population reporting alcohol intake patterns indicative of risky drinking. Female nurses in age group 25–34 years, 35–44 years and 45–54 years had significantly higher proportions of risky drinking than their counterparts in the general population, ranging from 6% to 12.8%, *p <* 0.001. The female Australian general population demonstrated a clear trend of increasing risky drinking with age [24], whilst for female nurses the trend appeared reversed; trends were similar but less marked for males. At 23.9%, nurses in the youngest age group (25–34 years) had rates of risky drinking which were significantly higher (*p <* 0.001) than nurses at ages 35–44 years (19.3%), 45–54 years (18.2%) and 55–64 years (14.6%). 

#### 3.2.5. Daily Smoking

**Figure 7:**
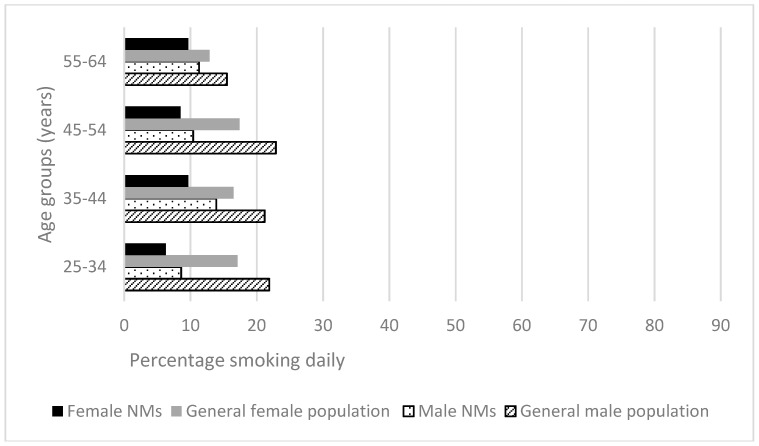
Health-related behaviors of nurses and midwives compared to the Australian general population 2014–2015 [30]: Smoking.

For both genders and at all ages nurses were less likely to smoke compared to the Australian general population [30]. Differences in proportions of male smokers amongst nurses compared to the general population ranged from 5.8% to 13.9% fewer, and were significant for all age groups except the small group aged 55–64 years. Differences in proportions of female smokers amongst nurses compared to the general population ranged from 2.7% to 7% (all *p <* 0.001). Overall, 81% of male and 87% of female nurses had never smoked compared to 53% of male and 63% of female members of the Australian general population; 8.6% of female and 10.8% of male nurses were current daily smokers compared to 16.9% of male and 12.1% of female members of the Australian general population [31] (Figure 7).

## 4. Discussion

Overall, this study presents a picture of nurses, and particularly younger nurses, at high risk for NCD due to diets poor in essential components, inadequate physical activity, high levels of overweight/obesity, central adiposity and risky drinking. Fortunately, relatively few were daily smokers. In several areas, nurses’ health behaviors indicated higher risk than that of the general population, including for inadequate physical activity and risky drinking, and central adiposity for females. However, fruit and vegetable intakes, whilst poor, were marginally better; smoking and male central adiposity was somewhat less prevalent in nurses.

These findings are cause for concern at nurses’ health-related behaviors and risk of NCDs, and are perhaps surprising, given nurses’ role in health promotion and presumed health literacy. This raises the possibility that work requirements and workplace conditions may impact health behaviors. The workplace has been flagged as a promising environment to deliver health interventions [32], yet at the same time many workplace-based barriers to healthy behaviors have been identified for nurses, including adverse work schedules and aspects of the physical workplace environment.

Multiple challenges impact food and eating habits: unhealthy workplace social eating practices [33], problems of food availability (limited facilities to store, heat and eat home-cooked food; time and distance barriers to food purchase) and variable quality (vending machines predominantly supplying junk food; cafés with menus of questionable food quality and facilities not conducive to staff comfort) have been identified [34]. This topic has been addressed with numerous intervention studies conducted to promote healthy eating for the general population and patients [35,36]. A review of intervention components concluded that dietary interventions are capable of producing meaningful weight loss both in the short and long term [35]; these are potentially of benefit for nurses, where obesity and central adiposity also predominate. However, interventions for the nursing workforce are scarce [37], indicating an area for future intervention, particularly targeting younger nurses.

In common with the general population, obesity and central adiposity increase with age in nurses. The older age profile of the nursing workforce makes this a particular concern. Moreover, compared with the young female general population, young female nurses were more likely to be obese and have central adiposity. These findings may be partly explained by our findings of young female nurses with worse eating and physical activity behaviors. This is a concern, as studies show that overweight/obesity and central adiposity increase the relative risk for NCDs, especially diabetes and coronary heart disease [38]. This is not the first study to raise concerns about overweight/obesity in this workforce; other studies have reported similar rates of high BMIs (e.g., 61.9% overweight/obese [39]).

More than half these nurses reported physical activity levels below recommendations. Previous studies have made similar observations and attributed this, in part at least, to shift work, particularly rotating and night shifts, which nurses blamed for restricting participation in leisure time activity [40]. We found that younger nurses were particularly less likely to meet the recommended physical activity levels. Exercise interventions combined with dietary changes have been shown to have synergistic benefits in relation to weight loss [41], prevention of diabetes, WC and blood pressure reduction [42]. However, high quality interventions to change nurses’ physical activity are inadequate and scarce [43].

Nurses reported substantial risky drinking, particularly amongst young male but also young female nurses, consistent with our previous study [21]. Studies of other Australian workforces show that, as the industry with the largest proportion of workers drinking at levels associated with harm [44,45], the nursing workforce (at 16.2%) has now overtaken hospitality (16.0%). In addition to health considerations, if working when alcohol-affected, risky drinking may also have serious professional effects in the workplace. Working under the influence of alcohol or when suffering hangovers, decision making may be impaired, posing a risk to patients and placing peers in difficult situations through, for example, mismanagement of medications or other unsafe or negligent practices [46]. This may result in damaged relationships with patients, actual harm or work injuries. Nurses who drink to excess may not be challenged about this due to fear of bullying, harassment or possible repercussions on their careers [47], thus missing an opportunity to access help. With risky drinking associated with alcohol-related absenteeism [45], findings also have implications for managers, such as increasing sick leave and budgets for casual payments.

Reasons for the use and abuse of alcohol by nurses have been considered [48]. Longer working hours have been shown to increase the likelihood of risky alcohol drinking by 11% [49]. Nurses’ work stress, physical, emotional or mental exhaustion and self-doubt [50] have potential effects on drinking habits; conversely, alcohol may be used as a stress management strategy [47]. Links between stress and alcohol use are well-recognized, but have been little studied in nurses. Whether the cause or result of stress, risky drinking poses direct negative effects for mental health, such as depression and anxiety [51].

Although relatively few nurses were daily smokers, 10.3% of nurses smoked and almost 15% of male nurses aged 35–44 years were daily smokers. Whilst nurse managers felt ‘stop smoking’ workplace interventions should not be a priority for this workforce due to the availability of such services in the community [52], the level of harm and associated ill-health and disease attributed to smoking and the (positive and negative) effects of health professionals’ role modelling of this behavior should prompt consideration of the cost-benefits of workplace-based effective quit smoking support.

Female workers predominate in the Australian nursing workforce. NCDs present the biggest threat to women’s health worldwide. For example, in Australia, 82% of women aged 55 and over have been diagnosed with osteoporosis or low bone density [53], and 49% of women aged between 65 and 74 years, as well as 63% aged 75 or above, with cardiovascular disease [54]. Reducing risk factors for NCDs should therefore be a priority for this workforce, which should benefit workforce productivity [4]. This study revealed patterns of particularly high risk amongst young nurses: for inactivity and risky drinking, and central adiposity in female nurses. This is a particular concern, for the patterns of behaviors established in the early career years and the implications for workforce retention [15].

Rapid increases in the prevalence of NCDs in the population place high demand on health services [5]. Nurses play an extremely important role in NCD prevention for patients [55] with many nurses providing health education to the public. Better management of nurses’ own health risk factors may benefit their health and may positively influence their willingness to engage with health promotion for their patients.

Whilst it has been suggested that nurses lack motivation to make lifestyle changes [56], the professional and environmental challenges and barriers encountered by the workforce have also been raised [19]. Given the nursing workforce context of shift work, long hours, and the stress and emotional labor of the occupation [19], nurses may find it difficult to make lifestyle changes, including within the workplace, without the support of their employers. Initiatives supporting health behaviors—diet, exercise, stress management and alcohol intake, and smoking—among nurses, perhaps particularly targeting younger nurses and rotating and night shift workers, are recommended. Policy-makers, senior managers, educationalists and researchers should consider strategies to both increase nurses’ motivation for lifestyle behavior changes and enhance the professional context and culture to facilitate and enable this.

This study has some limitations. This was a self-report survey, attended by the usual limitations of participant recall and potential challenges to response veracity. The sample was self-selected and whilst sample demographic characteristics resembled those of the national workforce [22], it is difficult to confirm its representative nature. Nurse numbers in the youngest (18–24 years) and oldest (≥65 years) age groups were relatively small, precluding comparisons with the Australian population across the whole workforce.

## 5. Conclusions

Nurses’ and midwives’ health is not only important within their own lives but also for the population who requires their services. Findings showing nurses, with high health literacy, with health behaviors no better than those of the population for whom they are role models is disappointing. This does perhaps reflect the pervasive influence of features of a modern environment that have been labelled as obesogenic. Within this, the influence of the workplace, occupied by fulltime staff for around one third of their waking lives, cannot be ignored.

With the ageing of the nursing workforce and their deleterious patterns of health behaviors occurring in tandem with increasing patient acuity, the increasing care needs of an ageing population, work stress and escalating workloads, effective intervention strategies for nurses are essential to prevent or delay increasing rates of NCDs in this workforce. With the potential advantages of the workplace flagged as a setting for health promotion, there are clearly opportunities to develop workplace-based interventions to promote the health of this workforce, particularly in the areas of physical activity and diet quality (which should impact weight and WC), alcohol intake and smoking cessation. The paucity of such interventions to date is a concern.

Managers should heed the health warnings of this study, and the clear need for supported workplace-based interventions to promote the health, productivity and longevity of the nursing and midwifery workforce. Health promotion strategies are urgently required that are suitable and feasible for delivery within the workplace, to safeguard the health and retention of this workforce and support their competence and credibility as role models and health educators of the population. A particular focus on young nurses is recommended, to address deleterious lifestyle health behavior patterns developing in the early career stages. Rigorous studies, including costed evaluation of such strategies, are required to enable rational decision-making for workforce health and productivity internationally.

## Figures and Tables

**Table 1:** Socio-demographic characteristics of nurses and midwives (*N* = 5041).

Characteristic	*N*	%
Age groups		
18–24	143	3.2
25–34	577	12.9
35–44	808	18.1
45–54	1400	31.4
55–64	1382	31.0
65 years and above	152	3.4
Gender		
Males	4421	90.6
Females	458	9.4
Education level		
Certificate/diploma	1245	27.8
Bachelor and above	3227	72.2
Work location		
Metropolitan areas	3313	66.5
Inner regional areas	1354	27.2
Outer regional and beyond	314	6.3
Work role		
Foundational	3560	71.7
Advanced practice	380	7.7
Domain-specific (manager, educator, researcher, etc.)	773	15.6
Work setting		
Hospital	3005	59.6
Aged care/rehabilitation or disability	686	13.6
Community centre/general practice/outpatients	896	17.8
Others	454	9.0
Working hours		
<40	2990	60.2
≥40	1975	39.8

**Table 2:** Associations between health behaviors, age and gender.

	**Odds Ratio (95% CI)**	***p* Value**
**Age Groups (years)**	**Vegetable Intake**	
25–34	1	
35–44	1.55 (1.04; 2.31)	0.03
45–54	1.79 (1.25; 2.58)	0.002
55–64	2.03 (1.41; 2.91)	<0.001
**Gender**		
Female	1	
Male	0.42 (0.26; 0.64)	<0.001
**Age Groups (years)**	**Fruit Intake**	
25–34	1	
35–44	1.08 (0.87; 1.34)	0.46
45–54	1.52 (1.25; 1.85)	<0.001
55–64	2.18 (1.79; 2.65)	<0.001
**Gender**		
Female	1	
Male	0.68 (0.56; 0.84)	<0.001
**Age Groups (years)**	**Physical Activity Level**	
25–34	1	
35–44	1.20 (0.97; 1.49)	0.09
45–54	1.19 (0.98; 1.44)	0.09
55–64	1.03 (0.85; 1.26)	0.75
**Gender**		
Female	1	
Male	1.28 (1.05; 1.58)	<0.02
**Age Groups (years)**	**Risky Drinking**	
25–34	1	
35–44	0.76 (0.59; 0.99)	0.04
45–54	0.71 (0.56; 0.90)	0.004
55–64	0.54 (0.43; 0.69)	<0.001
**Gender**		
Female	1	
Male	2.03 (1.61; 2.56)	<0.001
**Age Groups (years)**	**Daily Smoking**	
25–34	1	
35–44	1.62 (1.09; 2.42)	0.02
45–54	1.34 (0.92; 1.96)	0.13
55–64	1.54 (1.06; 2.23)	0.03
**Gender**		
Female	1	
Male	1.32 (0.95; 1.83)	0.09
**Age Groups (years)**	**BMI**	
25–34	1	
35–44	1.88 (1.50; 2.35)	<0.001
45–54	1.91 (1.55; 2.33)	<0.001
55–64	2.25 (1.83; 2.76)	<0.001
**Gender**		
Female	1	
Male	1.64 (1.31; 2.06)	<0.001
**Age Groups (years)**	**Waist Circumference**	
25–34	1	
35–44	1.59 (1.23; 2.07)	<0.001
45–54	2.04 (1.61; 2.59)	<0.001
55–64	2.68 (2.10; 3.40)	<0.001
**Gender**		
Female	1	
Male	0.31 (0.25; 0.39)	<0.001
